# Supplementary material for: Overexpression of Connexin 40 in the Vascular Endothelial Cells of Placenta with Acute Chorioamnionitis
Source: Diagnostics (Basel). 2024 Apr 12;14(8):811. doi: 10.3390/diagnostics14080811 (PMC11048802; doi:10.3390/diagnostics14080811)
Supplement: Supplementary file 1 [file diagnostics-14-00811-s001.zip › diagnostics-2913148-supplementary.pdf]

Supplementary Table S1. List of 42 cases of acute chorioamnionitis showing Cx43 and Cx40 expressions in various placental cell types and their perinatal outcomes.

No	Age	Race	Para	AG	Stages ACA		Perinatal outcomes				Connexin 43											Connexin 40										
					MIR	FIR	AS ≤3	IUD/ miscarriage	Prem	Lung cx	AEC	UVEC	UVSMC	CVEC	CVSMC	SVEC	SVSMC	CYT	SYNT	MVEC	DC	AEC	UVEC	UVSMC	CVEC	CVSMC	SVEC	SVSMC	CYT	SYNT	MVEC	DC
1	33	M	2-4	38	1	0	No	No	No	No	N	N	N	N	N	N	N	N	N	N	P	P	P	P	P	N	P	N	N	P	P	P
2	25	M	1	38	1	0	No	No	No	No	N	N	N	N	N	N	N	N	N	N	P	P	P	N	P	N	N	N	N	P	P	P
3	33	C	2-4	40	1	0	No	No	No	No	N	N	N	N	N	N	N	N	N	N	P	P	P	N	P	N	N	N	N	P	P	P
4	27	M	1	40	1	0	No	No	No	No	N	N	N	N	N	N	N	N	N	N	P	P	P	N	P	N	N	N	N	P	P	P
5	29	C	1	38	1	0	No	No	No	No	N	N	N	N	N	N	N	N	N	N	P	P	P	N	P	N	N	N	N	P	P	P
6	26	M	1	41	2	0	No	No	No	No	P	N	N	N	N	N	N	N	N	N	P	N	P	N	P	N	N	N	N	P	N	P
7	29	M	2-4	39	2	0	No	No	No	No	N	N	N	N	N	N	N	N	N	N	P	P	P	N	P	N	P	N	N	P	P	P
8	34	O	2-4	14	3	0	-	Yes	Yes	-	N	N	N	N	N	N	N	N	N	N	P	P	P	P	P	P	N	N	N	P	P	P
9	31	O	2-4	40	1	1	No	No	No	No	N	N	N	N	N	N	N	N	N	N	P	P	P	N	P	N	N	N	N	P	P	P
10	30	M	2-4	39	1	1	No	No	No	No	N	N	N	N	N	N	N	N	N	N	P	N	P	N	P	N	N	N	N	P	P	P
11	29	I	1	40	1	1	No	No	No	No	N	N	N	N	N	N	N	N	N	N	P	P	P	N	N	N	P	N	N	P	P	P
12	29	M	1	37	2	1	No	No	No	No	N	N	N	N	N	N	N	N	N	N	P	P	P	N	P	N	N	N	N	P	P	P
13	31	M	1	39	2	1	No	No	No	No	N	N	N	N	N	N	N	N	N	N	P	P	P	P	P	P	P	N	N	P	P	P
14	29	M	2-4	40	2	1	No	No	No	No	N	N	N	N	N	N	N	N	N	N	P	P	P	N	P	P	N	N	N	P	P	P
15	37	M	2-4	38	2	1	No	No	No	No	N	N	N	N	N	N	N	N	N	N	P	P	P	P	P	N	N	N	N	P	P	P
16	29	M	1	39	2	1	No	No	No	No	N	N	N	N	N	N	N	N	N	N	P	P	P	N	P	N	N	N	N	P	P	P
17	28	M	1	34	2	1	No	No	Yes	Yes	N	N	N	N	N	N	N	N	N	N	P	P	P	P	P	P	P	N	N	P	P	P
18	31	M	2-4	16	3	1	-	Yes	Yes	-	N	N	N	N	N	N	N	N	N	N	P	P	P	G	P	N	N	N	N	P	P	P
19	27	M	1	32	3	1	No	No	Yes	No	N	N	N	N	N	N	N	N	N	N	P	N	P	N	P	N	N	N	N	P	N	P
20	30	M	1	38	3	1	No	No	No	No	N	N	N	N	N	N	N	N	N	N	P	P	P	N	P	N	P	N	N	P	P	P

21	31	O	2-4	23	1	2	-	Yes	Yes	-	N	N	N	N	N	N	N	N	N	N	P	P	P	N	P	N	N	N	N	P	P	P
22	29	M	1	39	2	2	No	No	No	Yes	N	N	N	N	N	N	N	N	N	N	P	P	P	N	P	N	N	N	N	P	P	P
23	26	M	1	38	2	2	No	No	No	No	N	N	N	N	N	N	N	N	N	N	P	P	P	P	P	P	P	N	N	P	P	P
24	27	M	2-4	32	2	2	No	No	No	Yes	N	N	N	N	N	N	N	N	N	N	P	P	P	N	P	N	N	N	N	P	P	P
25	28	M	1	38	2	2	No	No	No	No	N	N	N	N	N	N	N	N	N	N	P	P	P	N	P	N	N	N	N	P	N	P
26	29	M	1	38	2	2	No	No	No	No	N	N	N	N	N	N	N	N	N	N	P	P	P	N	P	N	N	N	N	P	P	P
27	39	C	1	39	2	2	No	No	No	Yes	N	N	N	N	N	N	N	N	N	N	P	P	P	N	P	N	N	N	N	P	P	P
28	32	C	1	30	3	2	No	No	No	Yes	N	N	N	N	N	N	N	N	N	N	P	P	P	N	P	N	N	N	N	P	P	P
29	30	M	2-4	40	3	2	No	No	No	Yes	N	N	N	N	N	N	N	N	N	N	P	P	P	N	P	N	N	N	N	P	P	P
30	31	C	1	39	3	2	No	No	No	No	N	N	N	N	N	N	N	N	N	N	P	P	P	N	P	N	P	N	N	P	P	P
31	33	M	1	16	3	2	-	Yes	Yes	-	N	N	N	N	N	N	N	N	N	N	P	P	P	P	P	N	P	N	N	P	P	P
32	24	M	1	40	3	2	No	No	No	Yes	N	N	N	N	N	N	N	N	N	N	N	P	P	N	N	N	N	N	N	P	P	P
33	38	M	2-4	39	1	3	No	No	No	No	N	N	N	N	N	N	N	N	N	N	P	P	P	N	P	P	P	N	N	P	P	P
34	39	M	5+	36	2	3	No	No	Yes	No	N	N	N	N	N	N	N	N	N	N	P	P	P	P	P	N	P	N	N	P	P	P
35	24	C	1	34	2	3	No	No	Yes	No	N	N	N	N	N	N	N	N	N	N	P	P	N	N	P	N	P	N	N	P	P	P
36	27	M	1	39	2	3	Yes	No	No	No	N	N	N	N	N	N	N	N	N	N	P	P	P	P	P	N	P	N	N	P	P	P
37	26	M	1	40	3	3	No	No	No	No	N	N	N	N	N	N	N	N	N	N	P	P	P	N	P	P	P	N	N	P	P	P
38	26	M	1	39	3	3	No	No	No	No	N	N	N	N	N	N	N	N	N	N	P	P	P	N	N	N	N	N	N	P	N	P
39	33	M	2-4	40	3	3	No	No	No	No	N	N	N	N	N	N	N	N	N	N	P	P	P	N	N	N	N	N	N	P	N	P

Para– parity, GA– gestational age, ACA– acute chorioamnionitis, MIR– maternal inflammatory response, FIR– foetal inflammatory response, AS– Apgar score, IUD– intrauterine death, Prem– prematurity, Lungcx– lung complications, AEC– amnion epithelial cells, UVEC– umbilical vessels endothelial cells, UVSMC– umbilical vessels smooth muscle cells, CVEC– chorionic vessels endothelial cells, CVSMC– chorionic vessels smooth muscle cells, SVEC– stem vessels endothelial cells, SVSMC– stem vessels smooth muscle cells, CYT– cytotrophoblasts, SYNT– syncytiotrophoblasts, MVEC– maternal vessels endothelial cells, DC– decidual cells, M–Malay, C– Chinese, I– Indian, O– others, N– negative, P– positive.

Supplementary Table S2. List of 42 cases of non-acute chorioamnionitis showing Cx43 and Cx40 expressions in various placental cell types.

**No.**	**Age**	**Race**	**Para**	**GA**	**Connexin 43**											**Connexin 40**										
					**AEC**	**UVEC**	**UVSMC**	**CVEC**	**CVSMC**	**SVEC**	**SVSMC**	**CYT**	**SYNT**	**MVEC**	**DC**	**AEC**	**UVEC**	**UVSMC**	**CVEC**	**CVSMC**	**SVEC**	**SVSMC**	**CYT**	**SYNT**	**MVEC**	**DC**
1	32	MALAY	2-4	38	N	N	N	N	N	N	N	N	N	N	P	P	P	P	P	N	N	N	N	P	N	P
2	38	CHINESE	2-4	38	N	N	N	N	N	N	N	N	N	N	P	P	P	P	P	P	N	N	N	P	N	P
3	31	MALAY	2-4	40	N	N	N	N	N	N	N	N	N	N	P	P	P	N	P	N	N	N	N	P	P	P
4	30	MALAY	2-4	39	N	N	N	N	N	N	N	N	N	N	P	P	P	P	P	N	N	N	N	P	P	P
5	38	MALAY	5+	38	N	N	N	N	N	N	N	N	N	N	P	P	P	N	N	N	N	N	N	P	N	P
6	29	MALAY	2-4	37	N	N	N	N	N	N	N	N	N	N	P	P	P	P	P	N	N	N	N	P	P	P
7	31	MALAY	2-4	38	N	N	N	N	N	N	N	N	N	N	P	P	P	N	P	N	N	N	N	P	N	P
8	38	MALAY	5+	38	N	N	N	N	N	N	N	N	N	N	P	P	P	N	P	N	N	N	N	P	P	P
9	32	OTHERS	2-4	35	N	N	N	N	N	N	N	N	N	N	P	P	P	P	P	N	N	N	N	P	P	P
10	32	MALAY	2-4	37	N	N	N	N	N	N	N	N	N	N	P	P	P	N	P	N	N	N	N	P	P	P
11	27	INDIAN	1	38	N	N	N	N	N	N	N	N	N	N	P	P	P	P	P	N	N	N	N	P	P	P
12	29	MALAY	2-4	39	N	N	N	N	N	N	N	N	N	N	P	N	P	N	P	P	N	N	N	P	N	P
13	26	MALAY	2-4	35	N	N	N	N	N	N	N	N	N	N	P	P	P	N	P	N	N	N	N	P	N	P
14	30	MALAY	2-4	39	N	N	N	N	N	N	N	N	N	N	P	N	P	N	P	N	N	N	N	P	N	P
15	31	CHINESE	2-4	39	N	N	N	N	N	N	N	N	N	N	P	P	N	N	N	N	N	N	N	P	P	P
16	27	MALAY	2-4	39	N	N	N	N	N	N	N	N	N	N	P	P	P	N	P	N	N	N	N	P	P	P
17	22	MALAY	2-4	38	N	N	N	N	N	N	N	N	N	N	P	P	P	N	P	N	N	N	N	P	N	P
18	33	MALAY	2-4	39	N	N	N	N	N	N	N	N	N	N	P	P	N	N	N	N	N	N	N	P	N	P
19	31	MALAY	1	37	N	N	N	N	N	N	N	N	N	N	P	P	P	N	N	N	N	N	N	P	N	P
20	27	MALAY	2-4	38	N	N	N	N	N	N	N	N	N	N	P	P	P	N	P	N	N	N	N	P	P	P
21	34	OTHERS	2-4	36	N	N	N	N	N	N	N	N	N	N	P	N	N	N	N	N	N	N	N	P	N	P
22	29	MALAY	2-4	37	N	N	N	N	N	N	N	N	N	N	P	N	P	N	P	N	N	N	N	P	P	P
23	27	CHINESE	2-4	32	N	N	N	N	N	N	N	N	N	N	P	P	P	N	P	N	N	N	N	P	N	P
24	34	CHINESE	2-4	39	N	N	N	N	N	N	N	N	N	N	P	P	P	N	P	N	N	N	N	P	P	P
25	37	MALAY	2-4	37	N	N	N	N	N	N	N	N	N	N	P	P	P	P	P	N	N	N	N	P	P	P

26	34	MALAY	2-4	38	N	N	N	N	N	N	N	N	N	N	P	P	P	P	P	N	N	N	N	P	P	P
27	30	OTHERS	5+	40	N	N	N	N	N	N	N	N	N	N	P	N	P	P	P	N	N	N	N	P	P	P
28	31	MALAY	2-4	38	N	N	N	N	N	N	N	N	N	N	P	P	P	N	P	N	N	N	N	P	P	P
29	24	MALAY	2-4	37	N	N	N	N	N	N	N	N	N	N	P	P	P	P	P	N	N	N	N	P	P	P
30	29	MALAY	2-4	39	N	N	N	N	N	N	N	N	N	N	P	N	P	N	P	N	N	N	N	P	P	P
31	31	MALAY	2-4	39	N	N	N	N	N	N	N	N	N	N	P	P	P	N	P	N	N	N	N	P	P	P
32	35	MALAY	2-4	37	N	N	N	N	N	N	N	N	N	N	P	P	P	N	P	N	N	N	N	P	P	P
33	38	MALAY	2-4	36	N	N	N	N	N	N	N	N	N	N	P	P	P	N	P	N	N	N	N	P	P	P
34	38	MALAY	5+	37	N	N	N	N	N	N	N	N	N	N	P	P	P	N	P	N	N	N	N	P	P	P
35	31	MALAY	2-4	38	N	N	N	N	N	N	N	N	N	N	P	P	P	N	P	N	N	N	N	P	N	P
36	35	MALAY	2-4	38	N	N	N	N	N	N	N	N	N	N	P	P	P	N	P	N	N	N	N	P	N	P
37	31	MALAY	2-4	40	N	N	N	N	N	N	N	N	N	N	P	P	P	N	P	N	N	N	N	P	P	P
38	28	MALAY	1	38	N	N	N	N	N	N	N	N	N	N	P	P	P	N	P	N	N	N	N	P	P	P
39	37	MALAY	5+	38	N	N	N	N	N	N	N	N	N	N	P	P	P	N	P	N	N	N	N	P	P	P
40	33	MALAY	2-4	37	N	N	N	N	N	N	N	N	N	N	P	P	P	P	P	N	P	N	N	P	P	P
41	27	MALAY	1	40	N	N	N	N	N	N	N	N	N	N	P	P	P	P	P	N	N	N	N	P	P	P
42	26	MALAY	1	39	N	N	N	N	N	N	N	N	N	N	P	P	P	N	P	N	P	N	N	P	P	P

Para– parity, GA– gestational age, AEC– amnion epithelial cells, UVEC– umbilical vessels endothelial cells, UVSMC– umbilical vessels smooth muscle cells, CVEC– chorionic vessels endothelial cells, CVSMC– chorionic vessels smooth muscle cells, SVEC– stem vessels endothelial cells, SVSMC– stem vessels smooth muscle cells, CYT– cytotrophoblasts, SYNT– syncytiotrophoblasts, MVEC– maternal vessels endothelial cells, DC– decidual cells, N– negative, P– positive.
